# Case Report: Metastatic Signet-Ring-Cell Carcinoma of the Bladder From Breast Invasive Lobular Carcinoma Detected by Computed Tomography

**DOI:** 10.3389/fonc.2022.835487

**Published:** 2022-02-16

**Authors:** Xiaoxiang Jin, Haibin Tang, Hualin Chen, Gang Chen

**Affiliations:** Department of Urology, The First Affiliated Hospital of Chongqing Medical University, Chongqing, China

**Keywords:** signet-ring-cell carcinoma, bladder carcinoma, breast invasive lobular carcinoma, metastasis, CT manifestations, clinical diagnosis, multiple cancers, case report

## Abstract

Secondary bladder tumors are relatively rare among all bladder tumors, while bladder metastases from breast cancer have been rarely reported. Furthermore, signet-ring differentiation may appear in the metastases from a breast invasive lobular carcinoma regardless of whether the primary breast tumor had signet-ring cells, which may cause diagnostic uncertainty. We report a case of a 55-year-old female patient with diffuse bladder thickening as the chief complaint and no specific clinical manifestations. While the cystoscopy showed multiple scattered red protuberances, the biopsy suggested signet-ring-cell carcinoma. The gastroscopy results suggested poorly differentiated adenocarcinoma with signet-ring cells. Considering the patient’s history of invasive lobular carcinoma of the breast, chronic myeloid leukemia, and metastatic endometrial carcinoma from the breast, we performed an immunohistochemical analysis and the results indicated that signet-ring-cell carcinomas of the stomach and bladder originated from the invasive lobular carcinoma of the breast. We performed positron emission tomography/computed tomography and the results showed that there were multiple bone metastases already present. This was the first English case report of invasive lobular carcinoma of the breast metastasizing to the uterus, stomach, bladder, and bones with multiple signet-ring-cell variations. This study shares our reasons for misdiagnosing and opinions on diagnosing and treating for this kind of cases.

## Introduction

Primary bladder tumor is the most common tumor of the urinary system, while secondary tumors of the bladder are rare, accounting for less than 2% of all bladder neoplasms (1). The most common primary sites include the colon, prostate, and rectum, and the tumors from these sites mainly invade the bladder directly. Tumors from the stomach, lung, or breast invade the bladder with distant metastases (2). Secondary signet-ring-cell carcinomas (SRCCs) are rare. Herein, we report a case of a 55-year-old female patient with metastatic SRCCs in the stomach and bladder from an invasive lobular carcinoma (ILC) of the breast.

## Case Description

A 55-year-old female with no relevant medical history was diagnosed with ILC of the breast in November 2013. After preoperative neoadjuvant chemotherapy was administered twice, modified radical mastectomy was performed. The biopsy showed ILC of the breast (T_2_N_2_M_0_) with right axillary lymph node metastasis. After administering several regimens of postoperative chemotherapy and radiotherapy, the patient recovered gradually and was followed up regularly.

**Figure 1:**
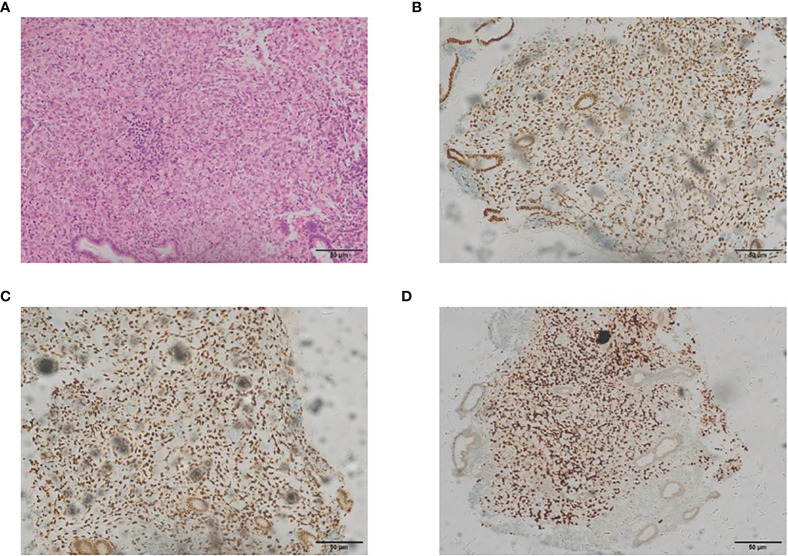
The representative staining results of endometrial carcinoma [**(A)** H&E, ×200; **(B)** estrogen receptor, ×200; **(C)** progesterone receptor, ×200; **(D)** GATA-3, ×200].

In May 2017, during a regular follow-up, the cancer antigen 125 level was found to be increased. The cervical and endometrial biopsies and immunohistochemistry analysis were performed. The expressions of the estrogen receptor (ER), progesterone receptor (PR) and GATA-3 in endometrial cells were positive, which showed that it was related to the metastatic endometrial carcinoma from the breast (**Figure 1**). Total hysterectomy and bilateral salpingectomy were performed after preoperative chemotherapy was administered twice, followed by four regimens of postoperative supplementary chemotherapy.

In May 2018, bone marrow aspiration was performed because of an unexplained rise in the platelet counts, and a diagnosis of chronic myeloid leukemia was made. The patient was then treated with imatinib (800 mg qd). In June 2020, the treatment was changed to flumatinib (600 mg qd) and in March 2021, it was changed to flumatinib (400 mg qd). The results of the P210/ABL were negative.

**Figure 2:**
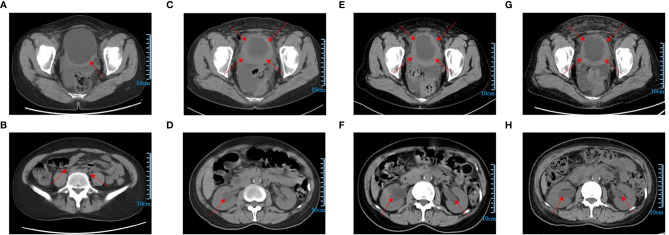
**(A)** The bladder wall thickened (August, 2020); **(B)** The bilateral ureter was slightly dilated (August, 2020); **(C)** The bladder wall was significantly thickened (March, 2021); **(D)** Hydronephrosis in the right kidney (March, 2021); **(E)** The bladder wall was significantly thicker than before (May, 2021); **(F)** Hydronephrosis in bilateral kidneys (May, 2021); **(G)** The bladder wall was still thick (September, 2021); **(H)** Hydronephrosis was slightly less than before (September, 2021).

**Figure 3:**
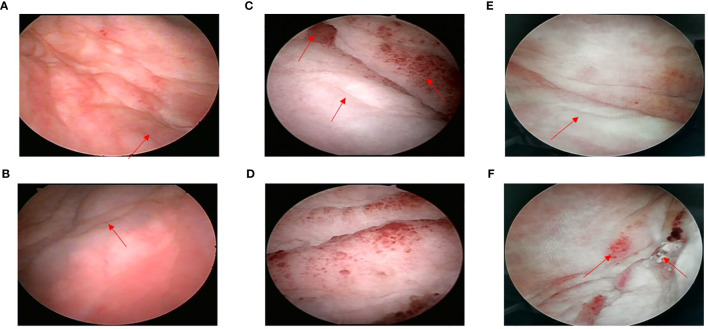
**(A)** Left ureteral opening. Bladder wall was locally thickening without new organisms (August, 2020); **(B)** Right ureteral opening (August, 2020); **(C)** Small red protuberances could be seen around left ureteral opening (May, 2021); **(D)** Right ureteral opening could not be seen (May, 2021); **(E)** Left ureteral opening (September, 2021); **(F)** Small red protuberances on bladder wall; right ureteral opening could not be seen (September, 2021).

During a routine follow-up with a computed tomography (CT) scan of the pelvic region in August 2020, it was found that the bladder wall had thickened, with the thickest part approximately 9.2 mm. The CT value was approximately 36 HU on a plain scan and 56 HU on an enhanced scan. The bilateral ureters were slightly dilated, and no obvious enlarged lymph nodes were found in the pelvic cavity. The uterus and bilateral appendages were not shown. There was a small amount of fluid in the pelvic cavity. The soft tissues of the abdominal and pelvic wall swelled slightly (**Figures 2A, B**). The cystoscopy showed that the appearance of the mucous membrane of each wall was normal with only a local thickening. No new organisms were found. The bilateral ureteral openings were normal. (**Figures 3A, B**). The patient experienced bladder distension after an injection of approximately 300 ml of normal saline. We considered it might be neurogenicbladder dysfunction at first. However, the patient had no high-risk factors such as diabetes or neurological diseases and she did not experience obvious hematuria, frequent urination, voiding pain, or other discomforts, and the creatinine and eGFR were normal (**Supplementary Figures 1A, B**). Therefore, we only suggested the patient active surveillance.

In March 2021, the pelvic CT showed a significant thickening of the bladder wall (**Figure 2C**). The CT value was approximately 39 HU on a plain scan and 47 HU, 63 HU, and 79 HU on enhanced scans in the arterial venous and delayed phases, respectively. The bilateral ureters were still slightly dilated, and no obvious enlarged lymph nodes were found in the pelvic cavity. The uterus and bilateral appendages were not shown and some small lymph nodes could be seen in the right inguinal region. There was a small amount of fluid in the pelvic cavity. The soft tissues of the abdominal and pelvic wall swelled slightly. However, unlike previously, there was hydronephrosis in the right kidney, and the pelvis antero-posterior diameter was estimated to be 22mm (**Figure 2D**). The patient still did not experience any chief urinary symptoms and the creatinine and eGFR were normal (**Supplementary Figures 1A, B**). This time, we still didn’t realize it was malignant. Because based on our previous experience, bladder cancer is more likely to show new organisms protruding into the cavity while bladder wall thickening is mostly related to non-cancerous lesions. And considering there were only slowly aggravated thickening of the bladder wall and the renal function was normal the whole time, we still cautiously suggested her active surveillance without placements of ureteral stents or nephrostomy tubes. Because for the patients who have histories of multiple tumors with poor physical conditions, sometimes active interventions may have a potentially negative impact in them.

In May 2021, the pelvic CT showed that the bladder wall was significantly thicker than previously observed. The CT value was approximately 39 HU on a plain scan and 45 HU, 64 HU, and 74 HU on enhanced scans in the arterial, venous, and delayed phases, respectively. The bilateral ureters showed a greater dilation than previously observed, and there was hydronephrosis in the bilateral kidneys. The pelvis antero-posterior diameter was estimated to be 21mm in right kidney and 14mm in left kidney, respectively. No obvious enlarged lymph nodes were found in the pelvic cavity. There were no changes in right inguinal lymph nodes and pelvic effusion compared to last time, but the swelling soft tissues of the abdominal and pelvic wall disappeared (**Figures 2E, F**). Based on rapidly progressive aggravations of the bladder wall thickening in the CT manifestations, we considered that there was a possibility of malignancy and we suggested that a cystoscopy should be performed immediately even though there were no significant clinical symptoms with normal renal function. The cystoscopy showed the presence of some small red protuberances around the left ureteral opening. The bladder wall had a tough, leather-like appearance. The right ureteral opening could not be seen (**Figures 3C, D**). After injecting about 100 ml of normal saline, the patient experienced bladder distension. We advised the patient to performed the pathological biopsy, but she refused this time without telling us the reason. We speculated that it may be related to the economic burden or psychological stress because the patient suffered from multiple cancers for a long time. The patient was asked to follow up regularly.

**Figure 4:**
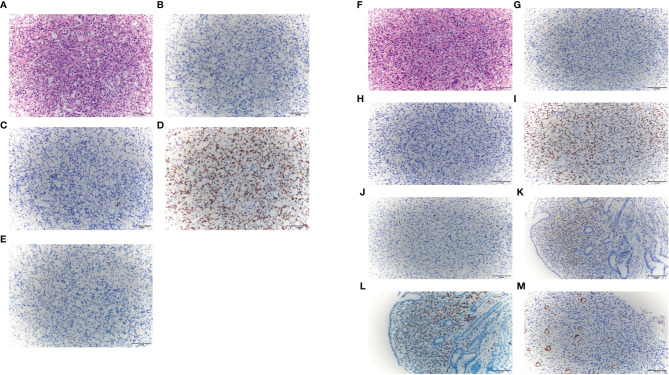
The representative staining results of bladder carcinoma [**(A)**. H&E, ×200; **(B)** CDX-2, ×200; **(C)** CK20, ×200; **(D)** GATA-3, ×200; **(E)** Villin, ×200]; The representative staining results of gastric carcinoma [**(F)** H&E, ×200; **(G)** CDX-2, ×200; **(H)** CK20, ×200; **(I)** GATA-3, ×200; **(J)** Villin, ×200; **(K)** estrogen receptor, ×200; **(L)** progesterone receptor, ×200; **(M)** E-cadherin, ×200].

Considering the prognosis may be poor without immediate intervention, we communicated with the patient many times. Finally, on May 24, 2021, the patient agreed to perform the second cystoscopy with a biopsy at the same time. The findings of the cystoscopy were similar to the previous findings, and a biopsy was performed this time. The pathological biopsy revealed the presence of signet-ring-cell carcinoma. An immunohistochemical analysis was performed. The results for caudal-type homeobox 2 (CDX-2), cytokeratin 20 (CK20), and Villin were negative, while the result for GATA-3 was positive (**Figures 4A–E**). Based on the histological findings and the previous diagnosis of breast ILC, a diagnosis of metastatic bladder SRCC from the breast was made. Gastroscopy and enterostomy were recommended. Gastroscopy showed there were hypertrophic protuberances of the mucous membrane in the fundus and body of the stomach, and the biopsy suggested poorly differentiated adenocarcinoma with signet-ring cells. The neoplastic cells were positive for ER, PR, and GATA-3 and negative for CDX-2, CK20, Villin and E-cadherin (**Figures 4F–M**). The results showed that the gastric carcinoma also originated from the breast. We performed positron emission tomography (PET)/CT scans, and the results showed that multiple bone metastases were already present.

Given the presence of multiple metastases, the patient was treated with apatinib mesylate (250 mg qd) and Tegafur Gimeracil Oteracil Potassium Capsule (40 mg bid). The patient returned to us on September 27, 2021, after three months of chemotherapy. She still experienced no typical urinary symptoms with normal renal function the whole time, and she had only a little gastrointestinal discomfort due to the chemotherapy. The CT scan showed that the bladder wall was still significantly thick. The CT value was approximately 38 HU on a plain scan and 45 HU, 62 HU, and 76 HU on enhanced scans in the arterial, venous, and delayed phases, respectively. The bilateral ureter dilation and hydronephrosis were slightly less than previously observed. The pelvis antero-posterior diameter was estimated to be 17mm in right kidney and 9mm in left kidney, respectively (**Figure 2G, H**). The cystoscopy showed there were still some small red protuberances on the bladder wall. The right ureteral opening could not be seen (**Figure 3E, F**). In the present case, chemotherapy appeared to be effective because for the patient with rapid progress at the beginning (from March 2021 to May 2021), there was no further aggravation after the chemotherapy. What’s more, the patient had no chief complaint in urinary system with normal renal function the whole time. and the patient was advised to follow up the renal function every month and the enhanced CT every 3months.

## Discussion

Metastatic lesions in the bladder represent less than 2% of all bladder neoplasms. Bladder metastatic tumors were mainly obtained from an autopsy series (1, 3, 4). Most of them reached the bladder by direct invasion from the prostate, female genital tract, and lower gastrointestinal tract. The other primary tumors originated from the stomach, breast, or lungs (2). About 45% of breast cancers have metastases, which can occur in almost any organ. Breast cancer often metastasizes to the bones, lungs, liver, and brain, known as organotropisms (5). Bladder metastases from breast cancer are extremely rare, with only about 65 cases reported (5, 6). The average time of urinary bladder metastases from the primary diagnosis of breast cancer is approximately 90 months (5). The main pathological subtypes of breast cancer are ductal and lobular carcinomas. ILCs, accounting for 8% to 14% of the cases, are the second most common subtype (6). Urinary bladder metastases from breast cancer are more common in ILC than in invasive ductal carcinomas (5–10).

Primary SRCCs of the bladder are uncommon, with an estimated prevalence of 0.24% of all primary bladder cancers (11, 12). Metastatic SRCCs of the bladder are rarer (12–24). We searched the PubMed, MEDLINE, Embase, and Google Scholar databases using key words such as “gastric or stomach”, “signet-ring-cell carcinoma”, “breast or mammary”, “bladder”, and “metastasis”. To the best of our knowledge, there have been no case reports of ILCs of the breast metastasizing to the uterus, stomach, bladder, and bones with multiple SRC variations.

For treatment, it is necessary to determine whether the tumor is primary or secondary. IHC is useful in differential diagnoses (25, 26). Recently, GATA-3 has been known as a specific marker for breast cancer, with almost 100% ILC of the breast expressing GATA-3, while the positive rate of GATA-3 expression may be less than 5% in primary tumors of the gastrointestinal tract and bladder (27). CDX-2 is a marker expressed only in normal gastrointestinal epithelial cells and tumors with more than two thirds of the cases of gastric adenocarcinomas expressing CDX2. However, in breast cancer, CDX2 is usually negative. Thus, it can also be used to identify tumors from the gastrointestinal tract or the breast (27). ER is expressed exclusively in breast carcinoma, but approximately 20% of breast SRCCs may be negative for ER (27, 28), whereas some studies have found that up to 30% of gastrointestinal adenocarcinomas were positive for ER. PR is also a common biomarker in breast carcinoma. In breast and ovarian adenocarcinoma, CK20 may often be negative. CK20 is positive in the gastrointestinal tumors (29–31). ER combined with CK20 and PR may be helpful in differentiating tumors that originate from the gastrointestinal region from those that originate in the breast (32, 33). Villin are produced mainly by epithelial cells that form brush borders. Villin-producing cells have been reported to be found in the epithelial cells of the intestinal mucosa and gallbladder. Thus, Villin may also be a useful marker for gastrointestinal cancer (34). It has been postulated that loss of E-cad expression is an early gatekeeper event in *in situ* lobular breast cancer and a precursor of invasive lobular breast cancer (35, 36). In our case, the bladder SRCC expressed GATA-3 and was negative for CDX-2, CK20, and Villin. The gastric SRCC expressed GATA-3, ER, and PR and was negative for CDX-2, CK20, Villin and E-cadherin. The endometrial carcinoma expressed ER, PR, and GATA-3. Therefore, we considered that all three metastatic tumors were from breast cancer.

However, the results of the immunohistochemical analyses are not always accurate. Diagnoses should be based on clinical manifestations, pathological biopsies, and imaging findings. For primary SRCCs, at present, surgical treatment is generally recommended, and it is possible for patients to have long-term disease-free survival (37). For secondary SRCCs, while chemotherapy is mainly used, the overall prognosis is poor. Our patient chose the chemotherapy regimen of apatinib mesylate (250 mg qd) and the tegafur gimeracil oteracil potassium capsules (40 mg bid). The chemotherapy appeared to be effective at the time because the hydronephrosis was slightly less than previously observed.

There are three special points in this case. First, the patient was not diagnosed accurately for approximately nine months, and when diagnosed finally, the PET/CT scans indicated bone metastasis. Generally, the manifestations of bladder metastatic tumors can be divided into two types: the protuberant type and the diffuse type. Almost all patients with gross hematuria show a protuberant mass during the imaging scans. In patients with lower urinary tract symptoms as the first symptom, the imaging findings are often diffuse wall thickening (12–23, 38). In our case, the pelvic CT scan showed the manifestation of the diffuse thickening of the bladder wall for nine months before the diagnosis of SRCC. However, no further treatment was made at that time because the patient showed no obvious clinical symptoms. Furthermore, the rarity of urinary bladder metastases made us relax our vigilance. We analyzed the following reasons for the missed diagnosis of the patient: 1) In general, the most common manifestations of bladder malignant tumors were hematuria, obvious space-occupying lesions, or obvious lower urinary tract symptoms (5). However, the urinary symptoms of this patient were not obvious all the time. This made us ignore the possibility of a tumor when there were only atypical bladder wall thickenings in the CT scans and negative findings from the cystoscopy, resulting in a missed diagnosis. 2) Metastatic bladder cancer is extremely rare, and it is often not included in the differential diagnosis when it is encountered with negative findings from the cystoscopy and with inconspicuous clinical symptoms. In fact, the metastatic pattern of ILC tends to occur as a diffuse thickening of the mucosa rather than as a discrete nodule (5). However, the subsequent progressive thickening of the bladder wall and the patient’s medical history of breast ILC should have prompted us to consider whether there was a risk of metastatic bladder cancer.

Second, there was a history of multiple cancers in this case. At present, the patient has a history of ILC of the breast, metastatic endometrial carcinoma from the breast, and chronic myeloid leukemia. Combined with the bone metastasis and the metastatic SRCC of the bladder and stomach, she had six types of cancers in different sites. There was no oncological disease in the patient’s family members, so we thought there may have been some mutations in the patient’s genes. We suggested the patient to do some genetic assessments, but the patient refused due to the high cost of genetic analysis. In the future, with the patient’s agreement, we intend to identify any related genes.

Third, there were no SRCs in the breast ILC; however, SRCs were found in the gastric and bladder metastases. SRC breast cancer was characterized in 1976 (39, 40). This type of tumor accounts for approximately 1% of all breast cancer cases (29, 39, 41). It is characterized by a particularly high proportion of signet-ring-shaped cells and is considered as a subtype of lobular carcinoma (39, 40, 42–47). Moreover, after searching the PubMed, Embase and Web of science databases, we found that SRCs may occur in metastases only and not in the primary tumors (39, 48). In other words, signet-ring differentiation may appear in the metastases from a breast ILC regardless of whether the primary breast tumor had SRCs, which may cause diagnostic uncertainty (39, 41, 43, 49). Due to this unique feature of breast ILC, in our opinion, if there is breast ILC combined with SRCC of other organs, we should consider whether there is a metastasis from the breast.

## Conclusions

Metastatic bladder SRCCs from breast ILCs are rare clinically. Atypical clinical symptoms and negative cystoscopy results often lead to a very high rate of missed diagnosis, which may delay treatment and affect the prognosis. Clinically, when we encounter patients that present with only atypical findings in CT scans such as bladder wall thickening, we should be careful about whether there is a possibility of metastatic bladder carcinoma, irrespective of whether the urinary symptoms and cystoscopy changes are typical, especially for those who have multiple cancer histories. Furthermore, when we encounter SRCCs in patients with histories of breast ILC, we should immediately consider whether there has been an SRC-type variation during the metastasis from breast ILC. Biopsies should be performed as early as possible and the immunohistochemical analysis is helpful in determining the primary lesion.

## Data Availability Statement

The original contributions presented in the study are included in the article/**Supplementary Material**. Further inquiries can be directed to the corresponding author.

## Ethics Statement

The studies involving human participants were reviewed and approved by the Medical Ethics Committee of Chongqing Medical University. The patients/participants provided their written informed consent to participate in this study.

## Author Contributions

XX contributed to the conception and design of the study. GC provided study materials or patients. HB and HL collected and assembled data. XX wrote the first draft of the manuscript. All authors contributed to manuscript revision, read, and approved the submitted version.

## Funding

The authors declare that this study received funding from the Senior Medical Talents Program of Chongqing for Young and Middle-aged from Chongqing Health Commission Program under Grant [number 2019GDRC017]. The funder was not involved in the study design, collection, analysis, interpretation of data, the writing of this article or the decision to submit it for publication.

## Conflict of Interest

The authors declare that the research was conducted in the absence of any commercial or financial relationships that could be construed as a potential conflict of interest.

## Publisher’s Note

All claims expressed in this article are solely those of the authors and do not necessarily represent those of their affiliated organizations, or those of the publisher, the editors and the reviewers. Any product that may be evaluated in this article, or claim that may be made by its manufacturer, is not guaranteed or endorsed by the publisher.

## Supplementary Material

Supplementary Figure 1
**(A)** The level of creatinine of this patient from August 2020 to December 2021; **(B)** The level of eGFR of this patient from August 2020 to December 2021.
