# Supplementary figures and images for: Case Report: Metastatic Signet-Ring-Cell Carcinoma of the Bladder From Breast Invasive Lobular Carcinoma Detected by Computed Tomography
Source: Front Oncol. 2022 Feb 16;12:835487. doi: 10.3389/fonc.2022.835487 (PMC8888881; doi:10.3389/fonc.2022.835487)

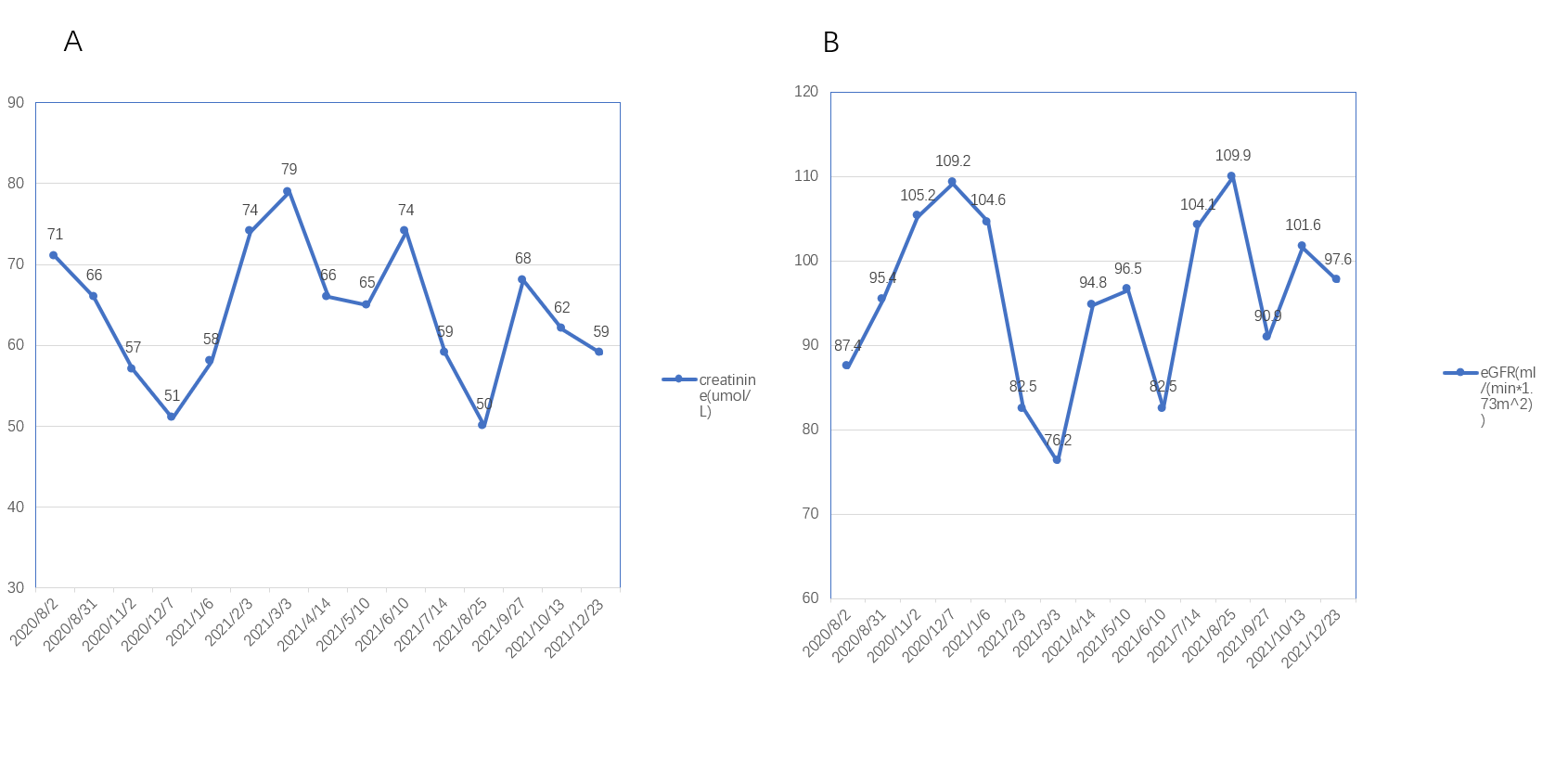

Supplement: Supplementary Figure 1 — (A) The level of creatinine of this patient from August 2020 to December 2021; (B) The level of eGFR of this patient from August 2020 to December 2021. [file Image_1.png]
